# Identification and Evolution Analysis of the Complete Methyl Farnesoate Biosynthesis and Related Pathway Genes in the Mud Crab, *Scylla paramamosain*

**DOI:** 10.3390/ijms23169451

**Published:** 2022-08-21

**Authors:** Ming Zhao, Fengying Zhang, Wei Wang, Zhiqiang Liu, Chunyan Ma, Yin Fu, Wei Chen, Lingbo Ma

**Affiliations:** Key Laboratory of East China Sea Fishery Resources Exploitation, Ministry of Agriculture and Rural Affairs, East China Sea Fisheries Research Institute, Chinese Academy of Fishery Sciences, Shanghai 200090, China

**Keywords:** methyl farnesoate, juvenile hormone, biosynthesis, methionine cycle pathway, *betaine-homocysteine S-methyltransferase* (BHMT), crustacean, *Scylla paramamosain*

## Abstract

The sesquiterpenoid hormone methyl farnesoate (MF) plays a vital role during crustacean development, which is mainly evidenced by its varied titers during different developmental stages. However, the biosynthesis pathways of MF remain obscure to some extent. In this study, we identified the complete MF biosynthesis and related pathway genes in *Scylla paramamosain*, including three involved in acetyl-CoA metabolism, eight in the mevalonate pathway, five in the sesquiterpenoids synthesis pathway, and five in the methionine cycle pathway. Bioinformatics, genomic structure, and phylogenetic analysis indicated that the JH biosynthesis genes might have experienced evolution after species differentiation. The mRNA tissue distribution analysis revealed that almost all genes involving in or relating to MF syntheses were highly expressed in the mandibular organ (MO), among which *juvenile hormone acid methyltransferase* was exclusively expressed in the MO, suggesting that most of these genes might mainly function in MF biosynthesis and that the methionine cycle pathway genes might play a crucial regulatory role during MF synthesis. In addition, the phylogenetic and tissue distribution analysis of the *cytochrome P450 CYP15-like* gene suggested that the epoxidized JHs might exist in crustaceans, but are mainly synthesized in hepatopancreas rather than the MO. Finally, we also found that *betaine-homocysteine S-methyltransferase* genes were lost in insects while *methionine synthase* was probably lost in most insects except *Folsomia candida*, indicating a regulatory discrepancy in the methionine cycle between crustaceans and insects. This study might increase our understanding of synthetic metabolism tailored for sesquiterpenoid hormones in *S. paramamosain* and other closely related species.

## 1. Introduction

The sesquiterpenoid juvenile hormones (JHs) play a vital role in arthropod development. To date, at least eight natural sesquiterpenoid hormones have been found in arthropod species, including JH 0,4-meth JH I (iso-JH0), JH I, JH II, JH III, JH III bisepoxide (JHB3), JH III skipped bisepoxide (JHSB3), and methyl farnesoate (MF) [1,2]. Among them, JH III and MF are the most common types in insects and crustaceans, respectively. MF is the precursor or non-epoxidized form of JH III, and the epoxidation of MF is catalyzed by a cytochrome P450 CYP15 enzyme [3], which was thought to only exist in insects [4]. However, results from recent studies indicate that a *CYP15-like* gene exists in crustaceans and might be related to MF metabolism [5]. In crustaceans, MF exhibits similar characters to JH III in insects, and is thought to be involved in the regulation of anti-metamorphosis [6,7,8] and ovarian development [9,10,11]. In addition, an in vitro study showed that MF and JH III show similar effects with regard to lipid accumulation in the crustacean hepatopancreas, but this effect was different from that of the MF precursor farnesic acid [12]. The regulatory role of JHs might be mainly controlled by the variation of titers during different developmental stages [13,14]. Therefore, it is of great importance to elucidate the biosynthesis and regulation pathways of MF.

In consideration of the general mevalonate pathway for acyclic isoprenoids [15,16] and JH biosynthesis in insects [17], the putative biosynthetic pathway for MF in crustaceans was thought to be undertaken by at least 12 enzymes, including acetoacetyl-CoA thiolase (AACT), HMG-CoA synthase (HMGS), HMG-CoA reductase (HMGR), mevalonate kinase (MevK), phosphomevalonate kinase (PMevK), mevalonate diphosphate decarboxylase (MDD), isopentenyl diphosphate isomerase (IPPI), FPP synthase (FPPS), farnesyl pyrophosphatase (FPPase), farnesol dehydrogenase-like (FoD), farnesal dehydrogenase (FaD), and juvenile hormone acid methyltransferase (JHAMT). MF biosynthesis was thought to be initiated by acetyl-CoA, which was catalyzed by a series of enzymes involved in the canonical mevalonate pathway to produce farnesyl pyrophosphate (FPP), then FPP was catalyzed following the arthropod specific pathway to produce MF [18]. Several putative key genes, including *AACT* [19], *HMGR* [20,21], and *JHAMT* [22,23,24,25], have been identified and characterized. However, the complete biosynthesis pathway for MF is still elusive for crustacean species. In addition, genes directly involved in the metabolism of acetyl-CoA as the starting material for MF biosynthesis, or S-adenosyl-L-methionine (SAM) as the methyl donor for MF, might also be important for MF biosynthesis. In fact, SAM is produced following the methionine cycle pathway, providing an active methyl group to numerous kinds of molecules, such as DNA, proteins, phospholipids, or neurotransmitters [26].

Early in 1987 and 1996, Laufer et al. [9] and Claerhout et al. [27] proved that the MF was secreted only in the mandibular organ (MO) in *Libinia emarginata* and *Homarus americanus*, respectively. MO was first identified and described in *Carcinus maenas* by Le Roux in 1968 [28], and subsequently identified in many other crustacean species [6,29]. MO is thought to be a homologous organ of the insect corpus allatum (CA), which synthesizes JHs in insects [6,29,30]. To date, it is still hypothesized that MO is the exclusive site for MF synthesis in crustaceans, and that MF is the only JH found in crustacean species. With the development of sequencing technology, several genome assemblies of crustacean species have been reported [31,32,33,34,35,36], providing valuable data for gene identification, species adaption, and evolution studies. For example, a comprehensive analysis of genes involved in sesquiterpenoid biosynthesis provided new insights into the spread of sesquiterpenoid hormones in the animal kingdom [37]. The characteristics and the mRNA distribution of these genes involved in sesquiterpenoid biosynthesis deserve further investigation.

Mud crabs comprise the genus *Scylla* (Portunidae, Decapoda, Crustacea), which includes four species, *S. olivacea*, *S. paramamosain*, *S. serrata*, and *S. tranquebarica* [38]. *S. paramamosain* is the most common mud crab in China [39]. Due to its rich nutritional value and delicious taste, the mud crab has high economic value, especially female crabs with mature ovaries. The physiological roles of MF are crucial for the economical traits of mud crabs. Previously, we reported a chromosome level genome assembly for *S. paramamosain* and the transcriptome data from both the Illumina and Pacbio platforms [36], which laid the foundation for the further identification of the complete pathway of MF biosynthesis in this species. Therefore, the overall goal of this research was to identify all the genes possibly involved in MF biosynthesis in *S. paramamosain* in order to provide an impetus for further research into sesquiterpenoid hormones.

## 2. Results

### 2.1. Identification of All Genes Putatively Involved in MF Biosynthesis

A summary of the genes putatively involved in sesquiterpenoid hormone biosynthesis is shown in Table 1, which includes 21 genes with 37 isoforms. The sequences of all *Scylla paramamosain* genes mentioned in this study are provided in Supplementary Table S2. The genes involved in the methionine cycle pathway are illustrated in Supplementary Figure S1. Among these genes, *AACT1*, *HMGS*, *HMGR*, and MS have two splicing isoforms, while two *AACTs*, two *FPPases*, seven *Fods*, three *betaine-homocysteine S-methyltransferases* (*BHMTs*), two *adenosyl homocysteinases* (*AdoHcyases*), and two *adenosine kinases* (*AKs*) have been found in *S. paramamosain*. In addition, *Fod2* is thought to be duplicated in the genome. The presence of two *HMGR* transcripts was reported in our previous study [20], and the complete mRNA sequences of *HMGR*, *FPPS*, *Fod1*, and *JHAMT* were validated using rapid amplification of cDNA ends (RACE) technology and Sanger sequencing.

**Figure 1:**
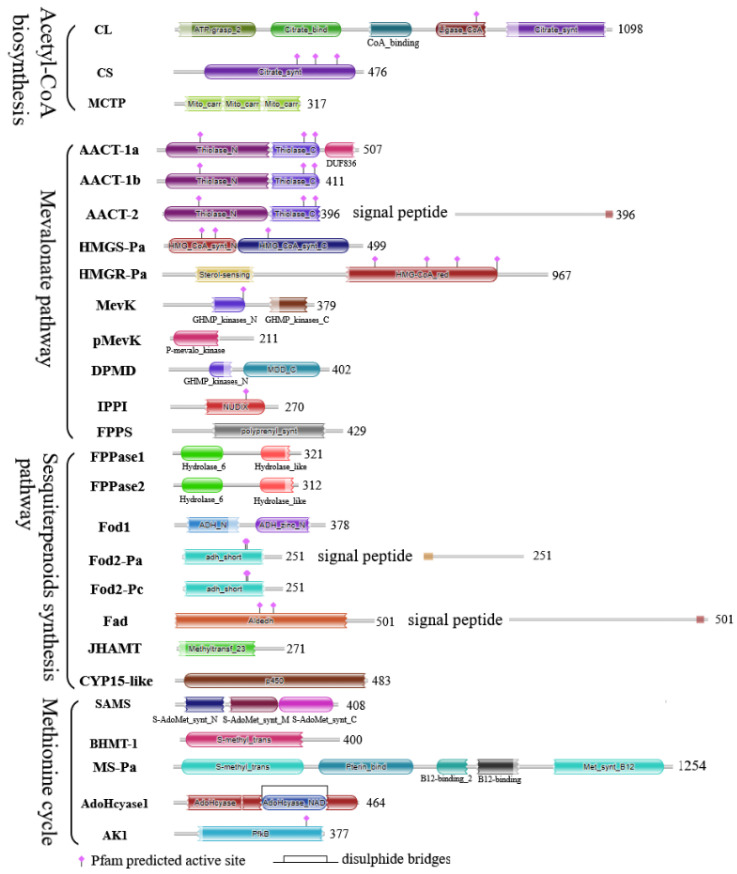
Protein domain analysis of MF synthesis or related pathway genes. The number on the right indicates the protein length; AACT-2 and Fad contained a predicted signal peptide on the C-terminal, and Fod2-Pa contained a predicted signal peptide on the N-terminal; AdoHcyase1 contained a predicted disulphide bridge.

The protein sequence identities of these genes between *S. paramamosain* and the *D. melanogaster* ranged from 34.158% to 84.265%. Among them, AdoHcyase 2 had the highest identity with *D. melanogaster* (84.265%), whereas mevK had the lowest (34.158%). The protein domains of these genes were analyzed using the HMMscan tool (Figure 1). Mevk protein sequences usually contained one GHMP_kinases_N and one GHMP_kinases_C domain, but the GHMP_kinases_C domain was absent in *D. melanogaster* and *B. mori*. The JHAMT protein sequence of *S. paramamosain* also had a low identity with that of *D. melanogaster* (37.681%), but the Methyltransf_23 domain, the SAM-binding motif, and the key catalytic Gln/His pair within *B. mori* JHAMTs [40] were conserved in those of *S. paramamosain* (Supplementary Figure S2). 

Additionally, a *CYP15-like* gene was also found in *S. paramamosain* that showed 34.34% and 39.88% protein identities with *D. melanogaster* CYP305a1 and *Diploptera punctata* CYP15a1 [3], respectively. 

### 2.2. Genome Structure of Genes Putatively Involved in MF Biosynthesis 

**Figure 2:**
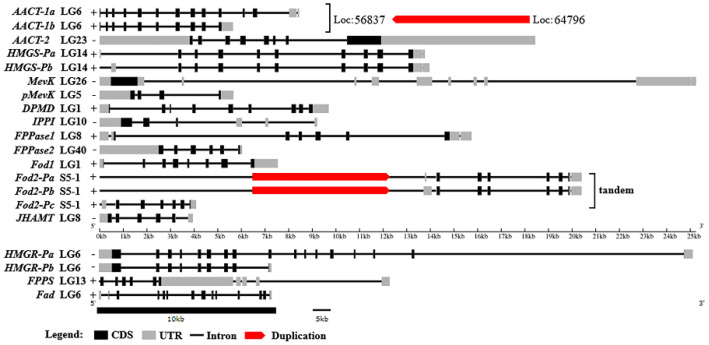
Genome structure of twelve genes putatively involved in MF biosynthesis. The IDs after the gene names indicate the pseudochromosome or scaffold IDs; + or—indicate positive and negative chains, respectively; red blocks indicate the direction of the gene duplication.

The genome structures of 16 genes are shown in Figure 2. Among them, only AACT1, HMGR and Fad were located in the same pseudochromosome (LG6), while the other genes were all located in different pseudochromosomes. A total of 11 genes were located in the forward strand and six genes were located in the reverse strand. All genes were split genes containing the intro sequences. The lengths of the exons ranged from 19 bp to 4154 bp, with an average length of 288 bp. *HMGR-Pa* was the longest gene, with a length of 149,443 bp, and contained 17 exons and 16 introns; *JHAMT* was the shortest gene, with a length of 3945 bp, and contained six exons and five introns. 

Additionally, we found that *AACT1* was duplicated in LG6, with another copy at the 64,796 bp downstream of the reverse strand. *Fod2* was duplicated in scaffold S5-1, with another two copies in the forward strand. The *Fod2-Pa* and *Fod2-Pb* were seemingly the spliced isoforms that were duplicated, while the *Fod2-Pc* had evolved and the identities of the mRNA and protein sequences between *Fod2-Pa* and *Fod2-Pc* were 57.2% and 88.0%, respectively.

### 2.3. Evolution Analysis of Sesquiterpenoid Biosynthesis Pathway Genes

**Figure 3:**
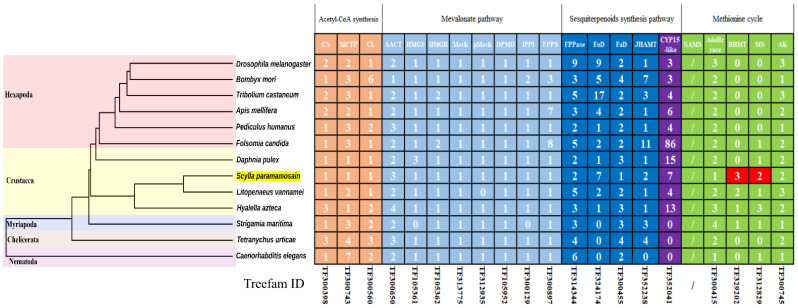
Statistics of genes involved in juvenile hormone biosynthesis among arthropods. The phylogenetic tree was constructed in our previous study; the numbers in the table indicate the gene numbers of the related gene family; statistical information on the SAMS gene family is not provided by TreeFam.

The numbers of each gene family are summarized in Figure 3. The results show that *S. paramamosain* had the complete MF biosynthesis pathway. Meanwhile, *CS*, *MCTP*, *FPPase*, *Fod*, *Fad*, *AdoHcyase* and *AK* genes were contracted in *S. paramamosain* when compared with the *D. melanogaster*, but these contractions were seemingly not crustacean specific, since some of these genes were of equal or greater number in other crustacea species when compared with hexapoda species. Interestingly, *BMHT* was seemingly lost in hexapoda, chelicerata, and nematoda species and only existed in three crustacea and myriapoda species with the exception of *Daphnia pulex*, while MS was seemingly lost in five of six hexapoda species with the exception of *Folsomina candida*.

**Figure 4:**
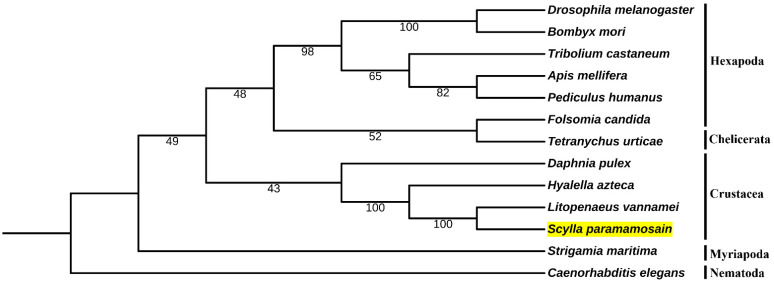
Phylogenetic tree constructed using seven genes involved in MF biosynthesis. The numbers on the branches indicate the bootstrap support; bootstrap values greater than 95% are indicated for clarity. The sequences of other species were downloaded from the NCBI genome database.

Seven genes with only a single copy in the *S. paramamosain* genome were tandem connected to construct a species phylogenetic tree (Figure 4). Our results show that this tree was generally in accordance with the species tree [36,41]. The differences include the position of *Tetranychus urticae*, a chelicerata species, which inappropriately clustered with a hexapoda species *Folsomia candida*. The position of *D. pulex* was only slightly different. However, these two branches had low bootstrap support. 

It was previously supposed that epoxidized JHs did not exist in crustacean species. However, the existence of a *CYP15-like* gene [5,42,43], which catalyzes the epoxidation of MF [3], and juvenile hormone epoxide hydrolase (JHEH) [44], which catalyzes the epoxide hydration of JHs, suggested that the epoxidized JHs may also existed in crustacean species. To make a further prediction, a phylogenetic tree using the protein sequences of the *CYP15-like* gene family was also constructed (Supplementary Figure S3). Our results show that CYP15A1, CYP15B1, CYP15, CYP305A1, and CYP303a1 were clustered in one big clade. 

To further clarify the phylogenetic relationship between gene families with more than one gene in *S. paramamosain*, we also constructed phylogenetic trees for the *AACTs*, *FPPases*, *Fods*, and *AdoHcyases* gene families (Supplementary Figures S4–S7). The results indicate that AACT genes can be divided in two types, the mitochondrial and cytosolic types, while AACT1 and AACT2 belonged to the mitochondrial and cytosolic type, respectively. However, the cytosolic type genes were not all clustered together in the tree. In addition, for other gene families, it seemed that those genes could not be clustered in several clades, indicating that these gene families might be involved in these species. 

### 2.4. Tissue Expression of MF Pathway Genes in Female Adult Crabs

**Figure 5:**
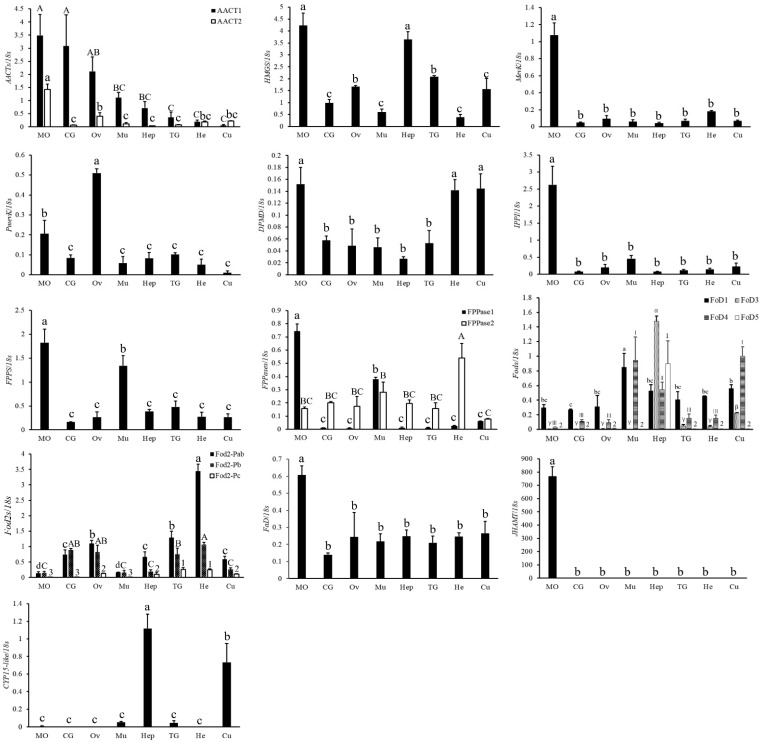
Expression profile of the mevalonate pathway and sesquiterpenoids synthesis pathway genes in different tissues of adult female crabs. The “Y” axis represents the relative ratio of the target genes/18S rRNA mRNA expression levels, and the gene names are provided in the ordinate title. The “X” axis represents different tissues in adult female crabs. Mandibular organ, MO; cerebral ganglion, CG; ovary, Ov; muscle, Mu; hepatopancreas, Hep; thoracic ganglia, TG; hemolymph, He; cuticle, Cu. The data are shown as the means ± SD (n ≥ 3). Different letters or numbers on the bar chart indicate significant differences (*p* < 0.05).

Since the mandibular organ (MO) is still thought to be the only organ that synthesizes MF in crustacean species, the mRNA distributions of all the above mentioned genes within different tissues were examined using qRT-PCR. Among the 13 genes involved in mevalonate and sesquiterpenoid synthesis pathway (Figure 5), all genes except *pMevK*, *Fod*, and *CYP15-like* had the highest expression in the MO; *pMevK* had the highest expression in the ovary (Ov), followed by MO; *AACT1* and *AACT2* both had the highest expression in the MO, while *AACT1* was also highly expressed in the cerebral ganglion (CG) and Ov; *FPPase1* had the highest expression in the MO, while *FPPase2* was ubiquitously expressed in all eight tissues. Compared with other *Fods*, *Fod1* had the highest expression in the MO; *JHAMT* was exclusively expressed in the MO. In addition, *CYP15-like* had the highest expression in the hepatopancreas (Hep), followed by cuticle (Cu), while it was rarely expressed in the MO, which was similar to *Portunus trituberculatus* [5], suggesting a conserved role of this gene in crustaceans (Figure 5).

**Figure 6:**
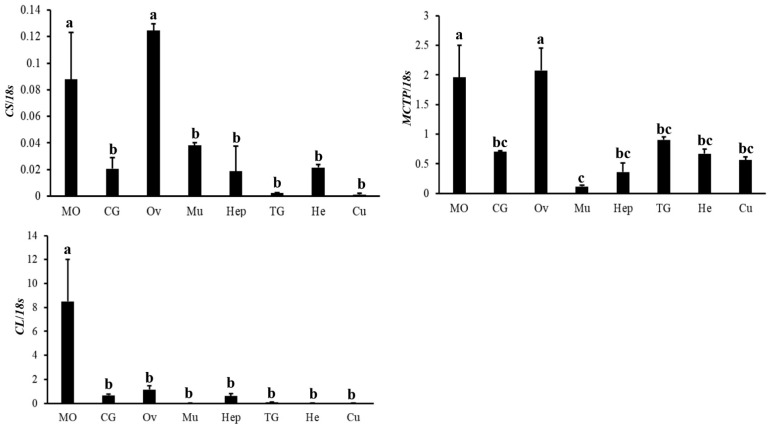
Expression profile of three genes involved in acetyl-CoA biosynthesis in different tissues of adult female crabs. The “Y” axis represents the relative ratio of the target genes/18S rRNA mRNA expression levels, and the gene names are provided in the ordinate title. The “X” axis represents different tissues in adult female crabs. Mandibular organ, MO; cerebral ganglion, CG; ovary, Ov; muscle, Mu; hepatopancreas, Hep; thoracic ganglia, TG; hemolymph, He; cuticle, Cu. The data are shown as the means ± SD (n ≥ 3). Different letters or numbers on the bar chart indicate significant differences (*p* < 0.05).

**Figure 7:**
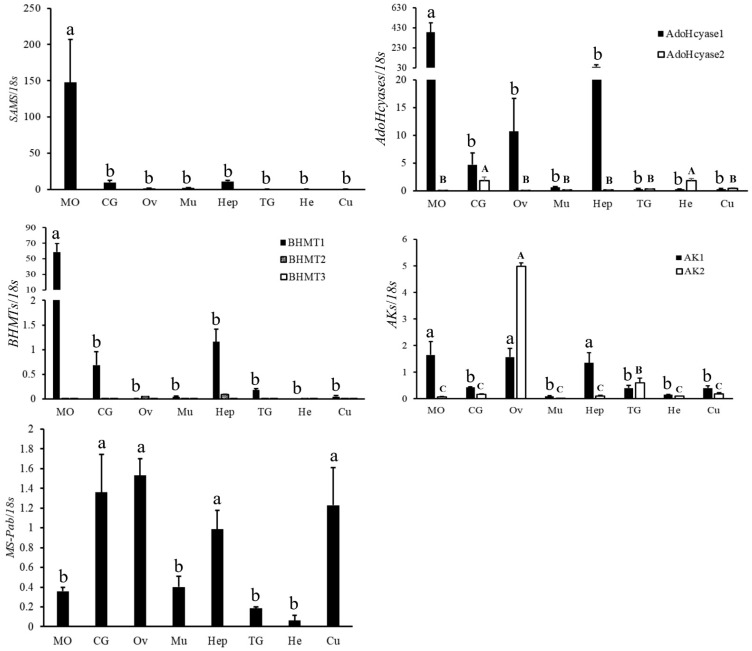
Expression profile of four genes involved in the methionine cycle in different tissues of adult female crabs. The “Y” axis represents the relative ratio of the target genes/18S rRNA mRNA expression levels, and the gene names are provided in the ordinate title. The “X” axis represents different tissues in adult female crabs. Mandibular organ, MO; cerebral ganglion, CG; ovary, Ov; muscle, Mu; hepatopancreas, Hep; thoracic ganglia, TG; hemolymph, He; cuticle, Cu. The data are shown as the means ± SD (n ≥ 3). Different letters or numbers on the bar chart indicate significant differences (*p* < 0.05).

The mRNA tissue distributions of genes involved in the metabolism of acetyl-CoA (Figure 6) or the methionine cycle pathway (Figure 7) were also examined. For genes involved in the metabolism of acetyl-CoA, *CS* and *MCTP* had similar expression patterns, showing the highest expression in the Ov and MO; *CL* had the highest expression in the MO, while it was less expressed in other tissues. In addition, for genes involved in the methionine cycle pathway, *SAMS*, *AdoHcyase1*, and *BHMT1* had the highest expression in the MO, which was ten times higher than in other tissue; *AdoHcyase2* was highly expressed in the CG and hemolymph (He); *BHMT2* and *BHMT3* showed low expression in all eight tissues; *AK1* had the highest expression in the MO, Ov, and Hep, while *AK2* had the highest expression in the Ov and lower expression in the MO; *MS* was highly expressed in the Ov, CG, and Cu and was less expressed in the Mu, MO, TG, and He.

## 3. Discussion

At present, it is still hypothesized that the MO is the exclusive site of MF synthesis in crustaceans and MF is the only JH found in crustacean species. Studies in insects have indicated that the accessory androgenic gland can also synthesize JH [45]. Although this phenomenon has not been observed in crustacean species, several MF synthesis genes have been shown to be expressed in tissues other than the MO [20,46]. In this study, we obtained the complete MF synthesis pathway genes, and we divided MF synthesis into four steps. The first step is the production of the starting material acetyl-CoA; the second step is the production of the FPP through the canonical mevalonate pathway using acetyl-CoA; the third step is the synthesis of MF through the arthropod-specific sesquiterpenoid synthesis pathway using FPP; the fourth step is the methionine cycle, which produces SAM as the meth donor for MF.

During the first step, since acetyl-CoA cannot pass through the mitochondrial membrane, CS catalyzes the reaction of acetyl-CoA (produced by glycolysis or other dissimilatory reactions) and oxaloacetic acid to produce citric acid and CoA; MCTP transports the citric acid from the mitochondria to the cytoplasm, then the citric acid is degraded by CL to produce acetyl-CoA in the cytoplasm as the starting material [47]. The identified CS, MCTP, and CL possess conserved protein domains and are highly expressed in MO, particularly for *CL*, which is less expressed in other tissues, indicating that the production of cytoplasmic acetyl-CoA in MO might be an important reaction. This is consistent with the finding that about 30% of glucose was used to synthesize JH in *Diploptera punctata* CA during an in vitro study [47]. 

During the second step, firstly acetyl-CoA molecules are aggregated by AACT. Two *AACTs* were identified in *S. paramamosain*, the mitochondrial type *AACT1* and cytoplasmic type *AACT2*. Both *AACT1* and *AACT2* had high expression levels in the MO, but *AACT2* was seemingly more specific in the MO. Clinical studies have shown that a lack of mitochondrial *AACT* causes severe ketoacidosis [48]. Highly expressed *AACT1* in the MO suggests that the MO might be an important organ for the reaction of acetyl-CoA. *HMGS*, *MevK*, *DPMD*, *IPPI* and *FPPS* all show the highest expression in the MO, which are consistent with the high expressions of these genes in the corpus allatum in insects [49,50] and the fact that *FPPS* is most highly expressed in the MO in *P. trituberculatus* [51]. In addition, two isoforms of *HMGS* have been found in insect species [52], and the fact that the two *HMGR* transcripts came from the same location was proven in this study [20].

The third step includes *FPPase*, *Fod*, *Fad*, and *JHAMT*. Two *FPPase*s and seven *Fods* were found in *S. paramamosain*. Both *FPPase1* and *Fad* were most highly expressed in the MO and were less expressed in other tissues, indicating that *FPPase1* and *Fad* might be the genes involved in MF synthesis. Among the seven *Fods*, *Fod1*, *Fod2-Pa*, and *Fod2-Pb* had a certain expression in MO, while others were rarely expressed in the MO, indicating that *Fod1*, *Fod2-Pa* or *Fod2-Pb* might be involved in MF synthesis. The phylogenetic tree of arthropod Fods showed that most Fods were clustered within species, indicating that *Fod* might have evolved after species differentiation, and which Fod is involved in MF synthesis could not be identified using the expression and phylogenetic analyses.

Additionally, there is some controversy regarding *JHAMT* in crustaceans. In 2002, Gunawardene et al. [53] reported that the in vitro expressed farnesoic acid methyltransferase (FAMeT) of *Metapenaeus ensis* could catalyze the conversion of farnesoic acid (FA) to MF. However, later in 2003 and 2004, Ruddell et al. [54] and Holford et al. [55], respectively, reported that the in vitro expressed FAMeT of *Cancer pagurus* or *Homarus americanus* had no catalyzing activity for FA. It was also noted that the mRNA of FAMeTs in many crustaceans were wildly distributed in various tissues [56,57,58], which was inconsistent with the hypothesis that the MO is the only MF biosynthesis organ. In 2014, Miyakawa et al. [24] reported an insect *JHAMT* ortholog in *Daphnia pulex*, and subsequently Toyota et al. [23] proved that JHAMT could catalyze the conversion of FA and that the catalyzing activity was similar to the insect *Tribolium castaneum*, while the catalyzing activity of *Macrobrachium rosenbergii* FAMeT [59] was only one-third that of *T. castaneum*. Further *JHAMTs* have also been found in *P. trituberculatus* [22] and *Neocaridina denticulata* [25]. *JHAMTs* in both *P. trituberculatus* and *S. paramamosain* are exclusively expressed in the MO, which is consistent with the hypothesis that the MO was the only organ for MF biosynthesis. In addition, knockdown of *P. trituberculatus JHAMT* significantly reduced hemolymph MF titers [22]. Furthermore, JHAMTs and FAMeTs contain different protein domains but the SAM binding site has only been identified in JHAMTs, and the reported catalysis activities of FAMeTs in vitro were all examined by radiochemical assay without a chromatography examination of the reaction products [53,59]. Therefore, we supported the idea that JHAMT is an enzyme that specifically catalyzes the FA to MF conversion in crustaceans, but the function of FAMeT still requires further investigation. 

During the fourth step, the methionine cycle pathway genes include *SAMS*, *AdoHcyase*, *BHMT*, *MS*, and *AK*. In insects, JHAMT catalyzes the methyl group transfer from SAM to juvenile hormone acid (JHA) to produce JH and S-Adenosyl homocystein (AdoHcy). However, AdoHcy can inhibit JHAMT activities, AdoHcy can be hydrolyzed by AdoHcyase, and the inhibitor of AdoHcyase can significantly inhibit JH biosynthesis [60]. The mRNA tissue expressions of *SAMS*, *AdoHcyase1*, and *BHMT1* are all highest in the MO, and the expression levels are similar to the those of *JHAMT* in the MO, suggesting that the methionine cycle might be important for MF biosynthesis. 

Another interesting finding was that the *BHMT* is lost in insects and that *MS* is partially lost in insects. *BHMT* only existed in three crustacean and one myriapoda species in this study. BHMT and MS both catalyze the conversion of homocysteine to methionine; BHMT transfers the methyl group from betaine while MS transfers the methyl group from methylated folic acid to homocysteine to produce methionine [26,61]. However, only *BHMT1* showed the highest expression levels in the MO, indicating that betaine and BHMT1 play more important roles in MF synthesis. In addition, substrate betaine is an important component of osmotic pressure regulation [62], and the metabolism of the product methionine might be related to lifespan [63], indicating that genes involved in the methionine cycle play a vital role during the life history of these organisms. In addition, it has been demonstrated that hyper-osmotic stress could elevate MF levels in several crustacean species [18]; whether BHMT is involved in this regulation deserves further exploration. 

Finally, phylogenetic trees constructed for a single gene family or the tandem connection of seven genes indicated a difference in the species differentiation [41], which were also found in the phylogenetic trees constructed by So et al. [37]. In addition, a comparison of the genomic structures of MF biosynthesis genes between *S. paramamosain* and two insects showed discrepancies between their genomic structures [49,64], suggesting that evolution events have occurred in the biosynthesis pathway. 

## 4. Materials and Methods

### 4.1. Ethics Statement

All animal experiments in this study were conducted in accordance with the relevant national and international guidelines. Our project was approved by the East China Sea Fisheries Research Institute. The mud crab *S.* *paramamosain* is not an endangered or protected species, and permission to perform experiments involving this species is not required in China.

### 4.2. Samples Collection 

The data used for the identification of genes involved in MF biosynthesis included genome assembly, Illumina-seq transcriptome, and Iso-seq transcriptome. For the Illumina-seq transcriptome, three MO samples, which were dissected from adult females at the early developing stage (stage II), the nearly ripe stage (stage IV) of the ovary, and on day 6 after the unilateral eyestalk ablated at stage II, were used; for the Iso-seq transcriptome, three mixed samples, including one mixture of different larval stages, one mixture of 20 tissues from male adult crabs, and one mixture of 20 tissues from female adult crabs, were used. All sample information mentioned above was described in our previous study, and the data were also deposited in a public database [36]. Eight tissues were dissected from nine female adult crabs and were used for mRNA tissue expression analysis using the quantitative real-time PCR (qRT-PCR) method.

### 4.3. Genes Identification

The identification of the genes involved in MF biosynthesis can be divided into at least three steps. First, we used a TreeFam ortholog [36] and the genes involved in the biosynthesis of JH in the fruit fly *Drosophila melanogaster*, the sequences of which were obtained from the KEGG database. For genes without TreeFam orthologs in mud crab, we used the local tblastN tool (blast+ Version 2.6.0) (with the parameters of e-value < e−10 and score > 100) to search for the genes with the highest identity to those of the fruit fly. Second, these mud crab genes were validated using the NCBI blastX tool to search the Non-Redundant Protein Sequence Database, and the conserved domains of the predicted protein sequences were also validated using the SMART [65] and HMMscan [66] tools. Third, the tissue distribution of all mRNA genes in adult crabs were validated using the qRT-PCR method, and the genes that were highly expressed in the MO were thought to be putatively involved in MF biosynthesis. In addition to these three steps, for genes with more than one copy in mud crabs, we also constructed a phylogenetic tree for further verification.

### 4.4. Sequence Accuracy Validation, Alternative Splicing and Genomic Structure Analysis

The sequence accuracies of these genes were validated using the Iso-seq and Illumina-seq transcriptome data. Using the local blastN tool, the Iso-seq and Illumina-seq date were used to verify the sequence structure and the nucleotide accuracy, respectively. In addition, all gene transcript sequences were mapped to the genome assembly using the Blat tool to identify the selective splicing events, and those sequences that could not be mapped were removed. The genome structures of these genes were exhibited using the online tool GSDS 2.0 [67].

### 4.5. Evolution Analysis

The multiple amino acid alignment of AACTs, FPPases, Fods, and AdoHcyases and the tandem connection of seven single-copy genes (including *HMGS*, *HMGR*, *MevK*, *DPMD*, *IPPI*, *FPPS*, and *JHAMT*) in twelve arthropod species and *Caenorhabditis elegans* were performed using MAFFT (v7.450) with the FFT-NS-I method [68]. The alignment of JHAMTs was also conducted using the DNAMAN software (version 8) to examine the conservation of key sites in mud crab.

The phylogenetic trees were obtained via the maximum likelihood method using IQ-tree (v1.6.12) [69] (1000 ultra-bootstrap replications and the best model detected by Modelfinder [70]). The final tree was polished with the online tool Interactive Tree of Life (https://itol.embl.de) (accessed from 1 January 2020 to 19 July 2022) [71].

### 4.6. Tissue Expression Analysis

Eight tissues, including hepatopancreas, ovary, cuticle, mandibular organ, cerebral ganglion, muscle, thoracic ganglia, and hemolymph, were collected by quick dissection, fixed in RNA fixer (TransGen Biotech, Beijing, China), and stored at −80 °C until the RNA extraction. RNA isolation, quality inspection, reverse transcription reaction, qRT-PCR system, and the procedure and quantitative method of qRT-PCR were described in our previous study [72]. The primers used in this study are provided in Supplementary Table S1. A relative standard curve was developed using 5-fold serial dilutions of cDNA. The standard curves were included in all runs to calibrate the quantitative data. The concentrations of cDNA in each sample were calculated from the standard curves.

To analyze the results of the qRT-PCR, the mean and standard deviation (SD) of each sample were calculated. All data obtained from the qRT-PCR analysis were log transformed prior to performing the data analysis with a one-way ANOVA. The post hoc test was carried out using a Tukey multiple comparison test. The differences were considered significant at *p* < 0.05. All data analyses were performed with SPSS 22.0.

## 5. Conclusions

This study identified 21 JH biosynthesis pathway genes in *Scylla paramamosain*, which included three acetyl-CoA metabolism genes, eight mevalonate pathway genes, five sesquiterpenoids synthesis pathway genes, and five methionine cycle pathway genes. Bioinformatics, genomic structures, and phylogenetic analyses indicated that the JH biosynthesis genes might have evolved after species differentiation. The mRNA tissue distribution analysis revealed that almost all genes that are involved in or relate to MF synthesis were highly expressed in the MO and that the *JHAMT* was exclusively expressed in the MO, suggesting that most of these genes mainly function in MF biosynthesis and that the methionine cycle pathway genes play an important regulatory role during MF synthesis. In addition, the phylogenetic and tissue distribution analysis of the *CYP15-like* gene suggested that epoxidized JHs might exist in crustaceans, but that they are mainly synthesized in the hepatopancreas rather than the MO. Finally, we found that *BHMT* genes were lost in insects and *MS* genes were partially lost in insects, indicating that a regulatory difference targeting the methionine cycle pathway exists between crustaceans and insects. This study has laid a good foundation for studies on the metabolism and functions of sesquiterpenoid hormones in closely related species.

## Figures and Tables

**Table 1:** Summary of identified MF synthesis or related pathway genes in *S. paramamosain*.

Gene Name	Abbreviation ^a^	Function	CDS Length (bp)	Identity with *Drosophila* Ortholog (%)
Acetyl-CoA metabolism genes				
Citrate (si)-synthase	**CS**	Synthesis of citrate in the mitochondria	1431	73.93
Mitochondrial citrate transport protein	**MCTP**	Transports citrate from mitochondria to cytosol	954	75.47
ATP citrate lyase	**CL**	Synthesis of cytosolic acetyl-CoA from citrate	3297	72.30
Mevalonate pathway genes				
Acetoacetyl-CoA thiolase	**AACT1-Pa**	Condenses two molecules of acetyl-CoA	1524	67.24
	**AACT1-Pb**		1236	67.24
	**AACT2**		1191	59.08
HMG-CoA synthase	**HMGS-Pa**	Condenses acetoacetyl-CoA + acetyl-CoA	1500	64.70
	**HMGS-Pb**		1500	-
HMG-CoA reductase	**HMGR-Pa**	Reduces HMG-CoA to mevalonate	2904	48.90
	**HMGR-Pb**		1965	-
Mevalonate kinase	**MevK**	Phosphorylates mevalonate	1140	34.16
Phosphomevalonate kinase	**pMevK**	Phosphorylates phosphomevalonate	636	45.60
Diphosphomevalonate decarboxylase	**DPMD**	Decarboxylates MPP to IPP	1209	51.37
Isopentenyl diphosphate isomerase	**IPPI**	Isomerization of IPP into DMAPP	813	52.96
Farnesyl diphosphate synthase	**FPPS**	Sequential condensation of IPP with DMAPP and then with GPP to form FPP	1290	50.55
Sesquiterpenoids synthesis pathway genes				
Farnesyl diphosphate pyrophosphatase	**FPPase1**	Hydrolysis of FPP to farnesol	966	/
	**FPPase2**		939	47.39
Farnesol oxidase	**FoD1**	Oxidation of farnesol to farnesal	1137	72.94
	**FoD2-Pa**		756	41.27
	**FoD2-Pb**		756	41.27
	**FoD2-Pc**		756	40.87
	**FoD3**		762	39.61
	**FoD4**		756	41.83
	**FoD5**		756	41.27
Farnesal dehydrogenase	**FaD**	Oxidation of farnesal to farnesoic acid	1506	57.44
Juvenile hormone acid methyltransferase	**JHAMT**	Transfers methyl group from SAM to farnesoic acid	816	37.68
Cytochrome P450 epoxidase	**CYP15-like**	Oxidation of MF into JH III	1452	39.88 ^b^
Methionine cycle pathway				
S-adenosylmethionine synthase	**SAMS**	Synthesis of SAM	1227	72.927
Adenosyl homocysteinase	**AdoHcyase1**	Hydrolysis of S-adenosyl-L-homocysteine	1395	78.241
	**AdoHcyase2**		1203	84.265
Betaine-homocysteine S-methyltransferase	**BHMT1**	Transfers methyl group from betaine to homocysteine to produce methionine	1203	\
	**BHMT2**		1092	\
	**BHMT3**		1107	\
Methionine synthase/5-methyltetrahydrofolate-homocysteine methyltransferase	**MS/MTR-Pa**	Transfer of a methyl group from methylated folic acid to homocysteine to produce methionine assisted by vitamin B12	3765	\
	**MS/MTR-Pb**		3777	\
Adenosine kinase	**AK1**	Hydrolysis of adenosine	1134	50.442
	**AK2**		1596	\

**Note:** ^a^. The genes that were identified in this study are in bold; ^b^. Identity with the *Diploptera punctata* CYP15a1; \ indicates that no orthologs were found in *Drosophila*;—indicates that this study did not provide statistical information.

## Supplementary Materials

The following supporting information can be downloaded at: https://www.mdpi.com/article/10.3390/ijms23169451/s1.
