# Supplementary material for: Identification and Evolution Analysis of the Complete Methyl Farnesoate Biosynthesis and Related Pathway Genes in the Mud Crab, Scylla paramamosain
Source: Int J Mol Sci. 2022 Aug 21;23(16):9451. doi: 10.3390/ijms23169451 (PMC9409210; doi:10.3390/ijms23169451)

Identification and evolution analysis of the complete methyl farnesoate biosynthesis and related pathway genes in the mud crab, *Scylla paramamosain*

Zhao *et al.*

Supplementary figures

Contents

Figure S1. The methionine cycle

Figure S2. Alignment of JHAMTs protein sequences

Figure S3. Phylogenetic tree of CYP15 gene family

Figure S4. Phylogenetic tree of AACTs gene family

Figure S5. Phylogenetic tree of FPPases gene family

Figure S6. Phylogenetic tree of Fods gene family

Figure S7. Phylogenetic tree of AdoHcyases gene family

Figure S1. The methionine cycle pathway. The figure was downloaded and modified from Ditscheid *et al.*, 2005 [26]. Genes identified in this study were underlined and the abbreviations were provided.

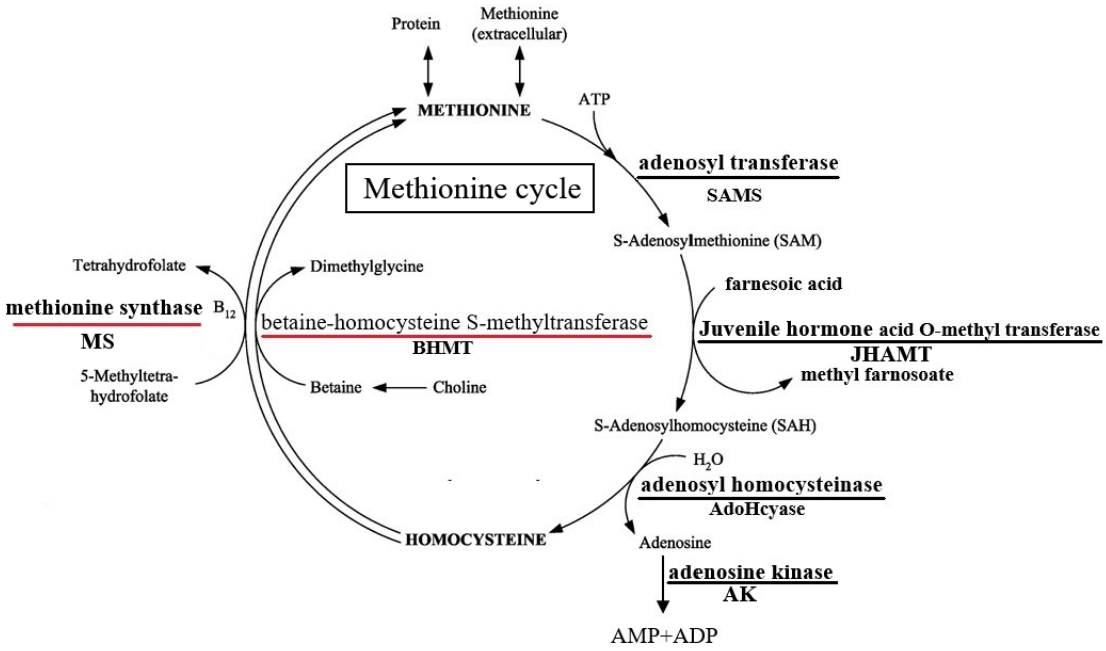

Figure S2. Alignment of JHAMTs protein sequences. The red box indicated the Methyltransf_23 domain; the green box indicated the SAM binding site D/EXGXG/AXG; two * indicated the two key catalytic Gln/His pair within *B. mori* JHAMTs [47].

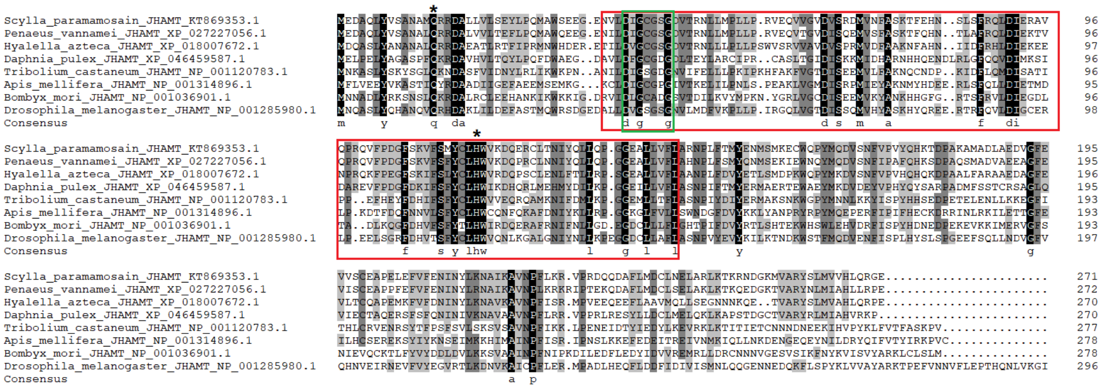

Figure S3. Phylogenetic tree of CYP15 gene family (Treefam ID: TF352041). The number on the branches indicate the bootstrap support, bootstrap values larger than 95% are indicated for clarity; *Bombyx mori* CYP306A1 was used as the root. Sequences of other species were download from NCBI genome database.

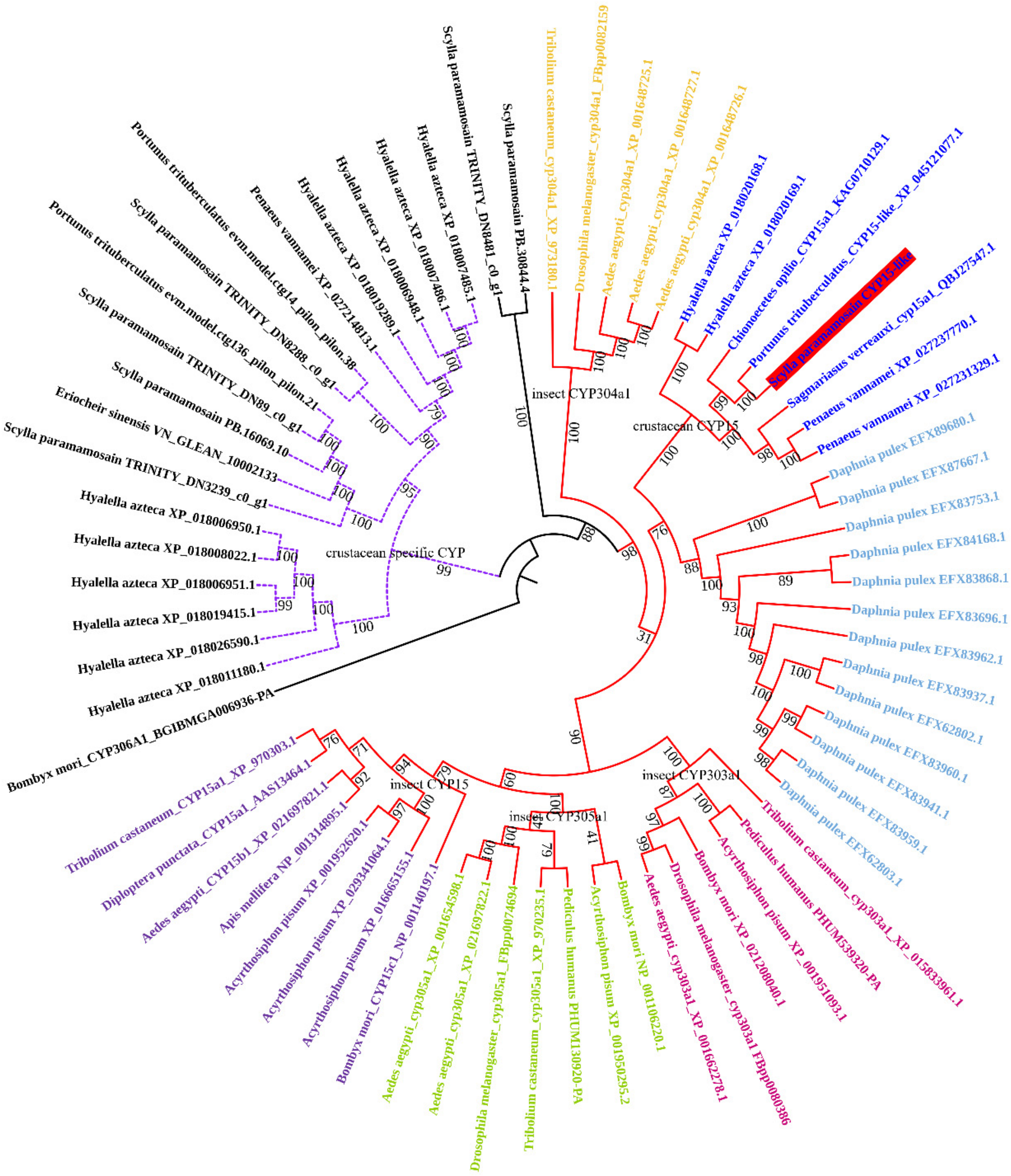

Figure S4. Phylogenetic tree of AACTs gene family (Treefam ID: TF300650). The number on the branches indicate the bootstrap support, bootstrap values larger than 95% are indicated for clarity. Sequences of other species were download from NCBI genome database.

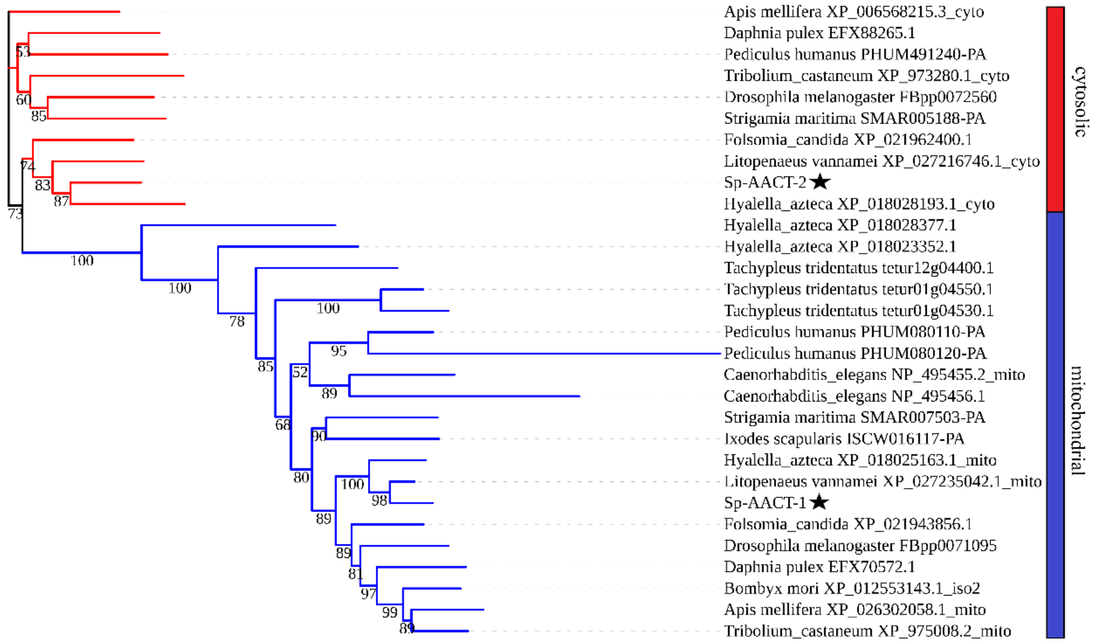

Figure S5. Phylogenetic tree of FPPases gene family (Treefam ID: TF314344). The number on the branches indicate the bootstrap support, bootstrap values larger than 95% are indicated for clarity. Sequences of other species were download from NCBI genome database.

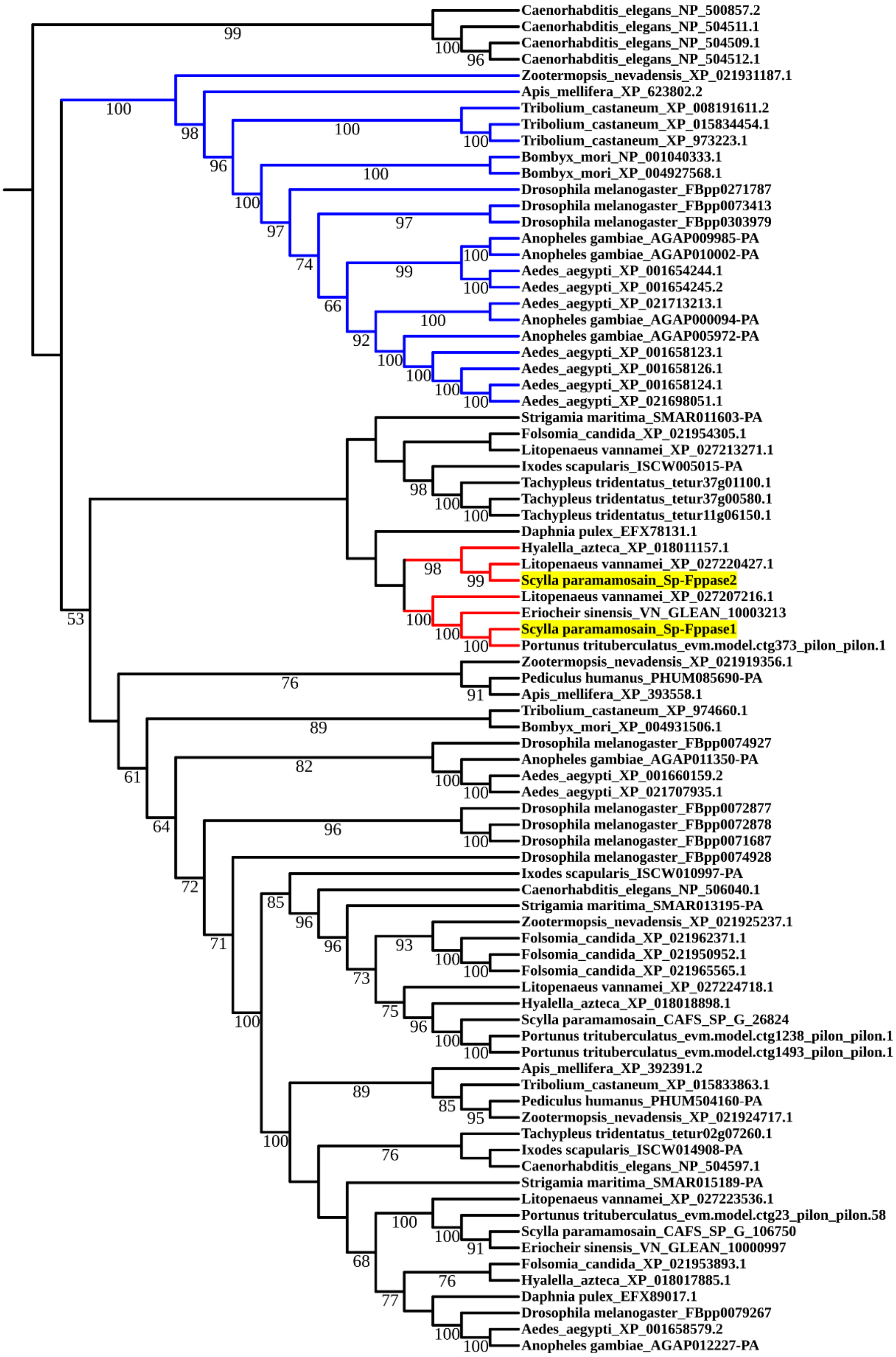

Figure S6. Phylogenetic tree of Fods gene family (Treefam ID: TF324174). The number on the branches indicate the bootstrap support, bootstrap values larger than 95% are indicated for clarity. Sequences of other species were download from NCBI genome database.

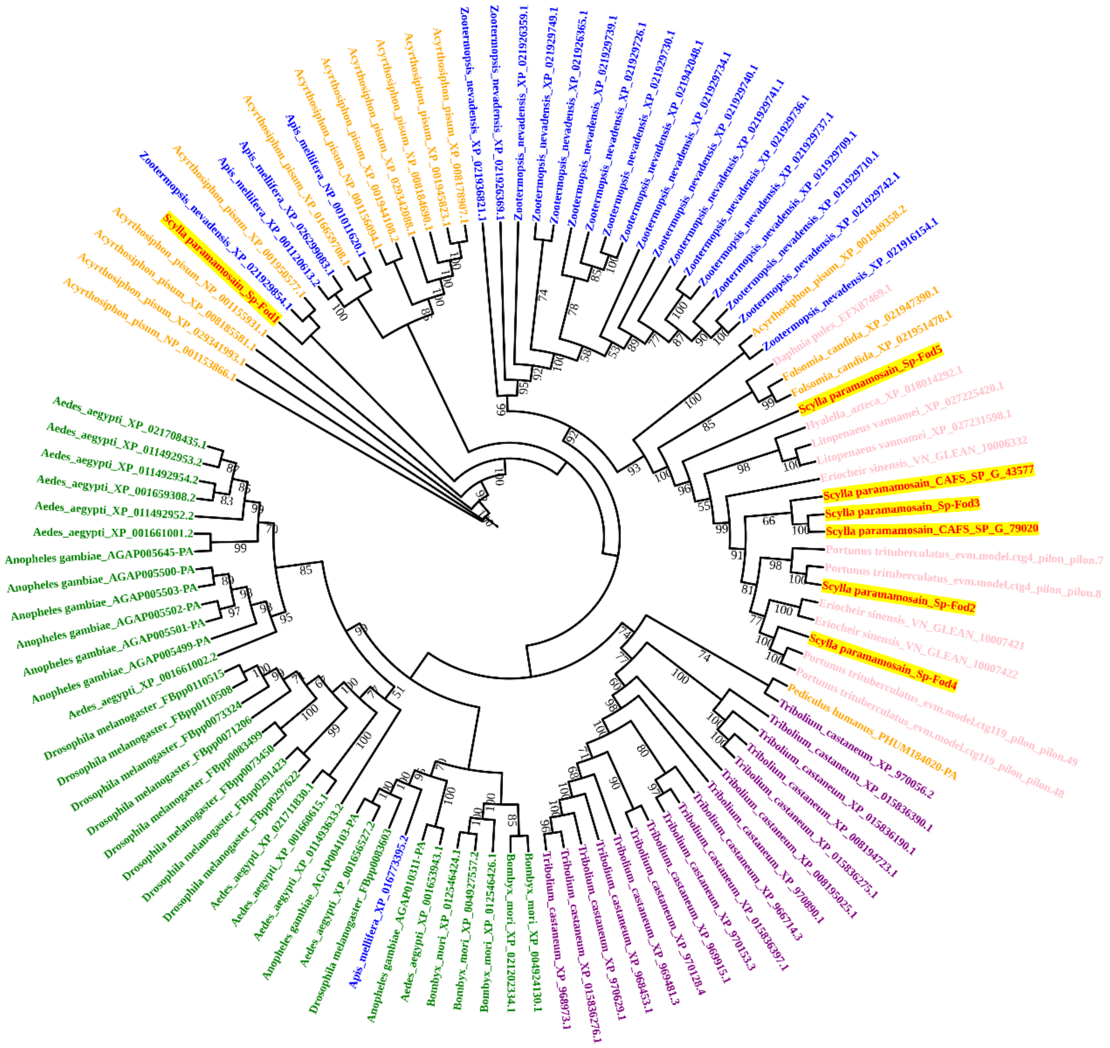

Figure S7. Phylogenetic tree of AdoHcyases gene family (Treefam ID: TF300415). The number on the branches indicate the bootstrap support, bootstrap values larger than 95% are indicated for clarity. Sequences of other species were download from NCBI genome database.

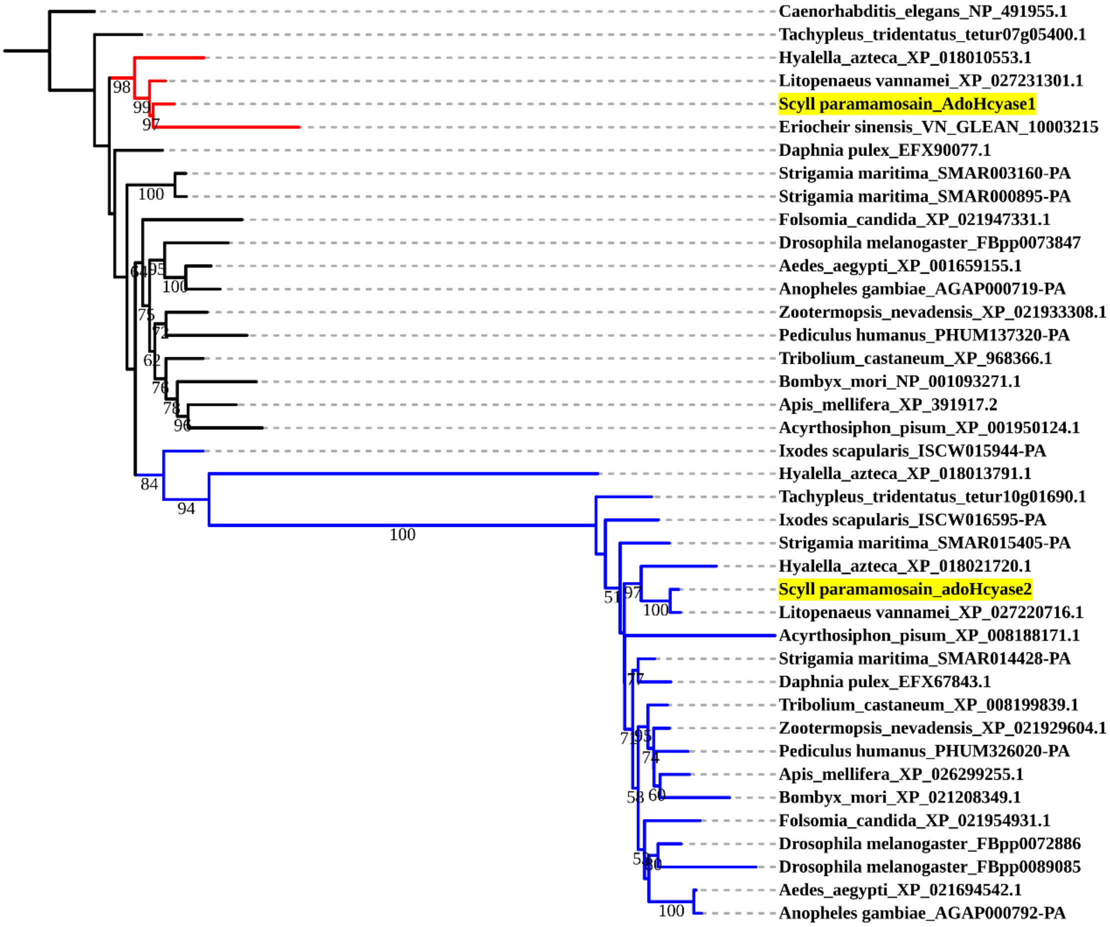

Supplement: Supplementary file 1 [file ijms-23-09451-s001.zip › ijms-1856304-supplementary/Supplementary figures.pdf]
